
JOURNAL INFORMATION
==============================
NLM Title Abbreviation: Int J Technol Assess Health Care
Journal ID: Int J Technol Assess Health Care
NLM Journal ID: 8508113
Journal ID: THC

International Journal of Technology Assessment in Health Care

ISSN: 0266-4623
EISSN: 1471-6348
Publisher: Cambridge University Press

ARTICLE INFORMATION
==============================
PMCID: PMC12766130
DOI: 10.1017/S0266462325100597
Article ID: S0266462325100597
Article version: 1
Subjects: Acknowledgements

Acknowledgements

Published online by Cambridge University Press

Publication date: 2025 Dec
Electronic publication date: 2025 Dec 29
Volume: 41
Issue: Suppl 1
Issue title: Abstracts from the HTAi 2025 Meeting in Buenos Aires, Argentina
First page: i
Last page: i
Copyright: © The Author(s) 2025
Copyright year: 2025
Copyright holder: The Author(s)
License: This is an Open Access article, distributed under the terms of the Creative Commons Attribution licence (https://creativecommons.org/licenses/by/4.0/), which permits unrestricted re-use, distribution, and reproduction in any medium, provided the original work is properly cited.
License URL: https://creativecommons.org/licenses/by/4.0/

Page count: 1

==============================
Acknowledgements

The HTAi Supplement 2025 showcases abstracts from the HTAi meeting in Buenos Aires, Argentina (14–18 June 2025). Thanks to Ann Scott and Jo Vabolis for editing this edition and for the support of HTAi staff.

